# Correlation of High-Risk Human Papilloma Virus with Deep Endometriosis: A Cross-Sectional Study

**DOI:** 10.1155/2023/6793898

**Published:** 2023-04-11

**Authors:** Zohreh Moslehi, Roya Derakhshan, Shahla Chaichian, Abolfazl Mehdizadeh Kashi, Babak Sabet, Samaneh Rokhgireh

**Affiliations:** ^1^Endometriosis Research Center, Iran University of Medical Sciences, Tehran, Iran; ^2^Faculty of Medicine, Shahid Beheshti University of Medical Sciences, Tehran, Iran; ^3^Smart University of Medical Sciences, Tehran, Iran

## Abstract

**Background:**

Recently, it has been suggested that microbial infections play a role in the pathogenesis of endometriosis. One of the most commonly suggested infections associated with the pathogenesis of endometriosis is human papillomavirus (HPV) infection. The present study is aimed at evaluating the prevalence, types, and risk factors for HPV infection in women with endometriosis and at investigating the association of upper and lower genital tract involvement with HPV and the severity of endometriosis.

**Methods:**

This cross-sectional study was conducted on 81 patients with endometriosis, referred to Rasool Akram Medical Complex in Tehran, Iran, for laparoscopic surgery. The patients' demographic, clinical, and anthropometric data were extracted from their medical records, as well as interviews. The stage of disease was scored based on the revised American Society for Reproductive Medicine (rASRM) classification. The HPV-positive and HPV-negative cases were compared using the chi-square test for categorical variables and Student *t*-test for continuous variables.

**Results:**

Twenty (24.69%) out of 81 women with endometriosis were infected with HPV (nine cases of pelvic HPV, nine cases of vaginal HPV, and two cases of both pelvic and vaginal HPV). The HPV-infected women had a significantly lower infertility rate (15% vs. 45.9%; *P* = 0.014). The VAS scores for dysmenorrhea and dyspareunia were relatively the same in the two groups (*P* > 0.05). HPV 6 and HPV 11 were the most common types of HPV, reported in 35% and 30% of endometriosis cases, respectively.

**Conclusion:**

The prevalence of HPV was 24.69%, and low-risk genotypes were dominant. No significant association was found between HPV and the severity of endometriosis.

## 1. Introduction

Endometriosis, which refers to the presence of endometrium outside the uterine cavity, can occur in the pelvis or outside the pelvic cavity (1). It is considered a common and complex gynecological disorder, with a prevalence of 2-17% (2). Although some patients with endometriosis have no symptoms, chronic pelvic pain, gastrointestinal symptoms, fatigue, and heavy menstrual bleeding may occur (3–6). Moreover, endometriosis has been reported in 30% to 50% of infertile women (7). Also, in some studies, an association has been suggested between endometriosis and the increased risk of ovarian cancer and extra-pelvic carcinomas (8).

Although the etiology of endometriosis is still under investigation, some findings suggest infertility, family history of endometriosis, and history of pelvic infection and uterine disorders as potential risk factors for endometriosis (9, 10). Lower genital tract infections have been also suggested as potential risk factors for endometriosis (11). In this regard, the results of a systematic review in 2020 showed that endometriosis is associated with the increased prevalence of Proteobacteria, Enterobacteriaceae, *Streptococcus*, and *Escherichia coli* bacteria across various microbiome sites (12). Evidence suggests that some sexually transmitted diseases caused by the human papillomavirus (HPV) and *Chlamydia trachomatis* are asymptomatic and may have severe reproductive tract sequelae. Also, the cervical microbiota is more diverse in women with HPV and *Chlamydia trachomatis* infections compared to healthy women. According to these findings, an association has been hypothesized between infections and endometriosis pathogenesis (13). Generally, HPV is one of the most common sexually transmitted infections and also one of the risk factors for cervical cancer. Because of the anatomical proximity of endometrium to the cervix, researchers are interested in the role of HPV in the development of endometrial cancer. The results of a meta-analysis in 2014 showed that the prevalence of HPV in endometrial cancer varied from 0% to 61.1%, with a pooled prevalence of 10% (95% CI: 5.2–16.2) (13, 14). However, considering the malignant and immunological processes of endometriosis, the possible role of infectious agents in the development of endometriosis has been recently proposed (15). Intrauterine infection can initiate endometriosis by activating the proinflammatory and innate immune pathways. Therefore, infection-associated chronic immune activation can facilitate the abnormal growth of endometrial tissue (16). Chronic peritoneal inflammation due to infection may activate natural killer cells and cause macrophage abnormalities. Complex mechanisms related to infection in the autoimmune pathway may contribute to the pathogenesis of endometriosis (17).

In recent years, studies have reported that microbial infections play a role in the pathogenesis of endometriosis. HPV is one of the most commonly suggested infections in the pathogenesis of endometriosis. Therefore, the present study is aimed at investigating the prevalence, types, and risk factors for HPV infection in women with endometriosis and at evaluating the association of upper and lower genital tract involvement with HPV and the severity of endometriosis.

## 2. Materials and Methods

This descriptive, analytical, cross-sectional study was conducted on 81 women with endometriosis, referred to Rasool Akram Medical Complex, affiliated to Iran University of Medical Sciences, Tehran, Iran, for laparoscopic surgery. All of the 81 patients underwent laparoscopic surgery for endometriosis. This study was carried out based on an estimated prevalence of 10-60% for HPV in the endometriosis.

(1) $$\begin{array}{c} N=\frac{N\;{Z_{1-\alpha /2}}^{2}\;p\left(1-p\right)}{d^{2}\left(N-1\right)+{Z_{1-\alpha /2}}^{2}\;p\left(1-p\right)}, \end{array}$$

where *P*_1_ = 0.4, *d*_2_ = 0.06, *Z*_1−*α*/2_ = 1.96, and *N* = 120.

The sample size was calculated from the following formula, and it was 81 people.

After describing the procedure to the participants, they were asked to sign an informed consent form. The inclusion criteria in this study were as follows: being a nonvirgin; age of 20-50 years; and a confirmed diagnosis of endometriosis based on the clinical signs, ultrasound findings, and surgical treatment if needed. On the other hand, insufficient sample for HPV testing was considered the exclusion criterion. The Ethics Committee of Iran University of Medical Sciences approved the protocol of this study (IR.IUMS.REC.1400.491).

Demographic, clinical, and anthropometric data of the patients, including age, body mass index (BMI), gravidity, parity, diagnosis time, uterus size, dyspareunia, dysmenorrhea, chronic pelvic pain, history of surgery, infertility, and menstrual status, were extracted from the patients' medical records, as well as interviews. Moreover, the stage of disease was scored by surgeons, based on the revised American Society for Reproductive Medicine (r-ASRM) classification. Deep endometriosis (DE) was considered a special form of endometriosis, infiltrating more than 5 mm below the surface of the peritoneum. Ovarian endometrioma (OMA) and the presence of nodules in the rectum, uterosacral ligament, and bladder were also recorded. Before the onset of laparoscopic surgery, a sample of the exocervix was collected from the cervical mucus to examine the presence of HPV, using a sterile Ayre spatula and a cytobrush (Cervex-Brush®, Rovers Medical Devices).

After the onset of surgery and determining the severity of endometriosis, to detect HPV in the cul-de-sac, a tissue biopsy was obtained from the uterosacral ligament by a surgeon and immediately placed in saline solution; after it was stored in a refrigerator at -20°C, it was sent to a laboratory. This tissue biopsy used for identifying HPV in the cul-de-sac was called pelvic HPV. The HPV Direct Flow CHIP was used for screening HPV types and genotyping HPV types by polymerase chain reaction (PCR) assay, followed by reverse dot-blot automatic hybridization based on the DNA Flow Technology (e-BRID System, Master Diagnóstica, Granada, Spain).

To describe the demographic and clinical characteristics of HPV-positive and HPV-negative patients, mean and standard deviation were measured for continuous variables and frequency and percentage for categorical variables. Fisher's exact test was performed for data analysis. The two groups were compared using the chi-square test for categorical variables and Student *t*-test for continuous variables. Data were analyzed in Stata 14 (Stata Corp. LLC, College Station, TX, USA). The significance level was considered to be less than 0.05.

## 3. Results

**Figure 1:**
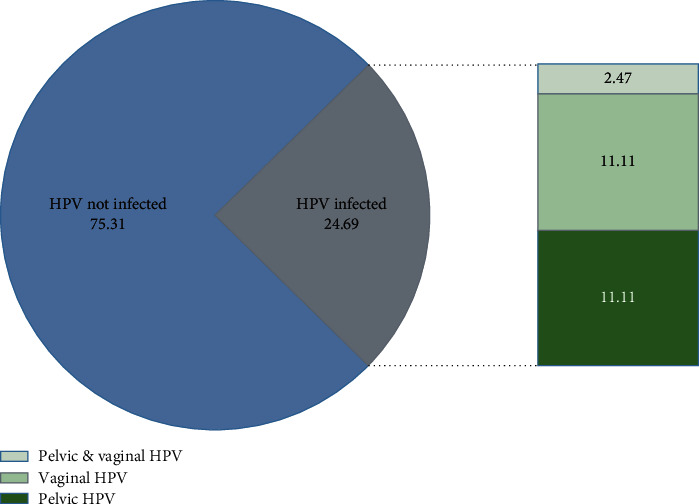
The proportion of HPV in women with endometriosis.

In this study, a total of 81 women with endometriosis were included. Twenty women (24.69%) were infected with HPV, nine women with pelvic HPV, nine women with vaginal HPV, and nine women with both pelvic and vaginal HPV (Figure 1).

In Table 1, some demographic, anthropometric, and clinical variables are compared between HPV-infected and HPV-noninfected cases. The two groups were homogenous regarding age, BMI, gravidity, parity, endometrioma size, cancer antigen 125 (CA125), and uterine size (*P* > 0.05). Although HPV-infected patients had higher rates of irregular menstrual cycles (45% vs. 40.98%) and rectal nodules (95% vs. 86.89%) and non-HPV patients had a higher rate of uterosacral involvement (82.25% vs. 70%), the differences were not statistically significant (*P* > 0.05). However, HPV-infected women had a significantly lower rate of infertility compared to noninfected women (15% vs. 45.9%, *P* = 0.014).

Overall, 31 patients (38.27%) were infertile (17.28% with primary infertility and 20.99% with secondary infertility).

The majority of non-HPV patients had secondary dysmenorrhea (96.72% vs. 75%; *P* = 0.003). The VAS scores for dysmenorrhea and dyspareunia were relatively the same in the two groups (*P* > 0.05) (Table 2).

Moreover, HPV-infected and HPV-noninfected women were compared regarding endometriosis management before the procedure. There was no significant difference between the two groups regarding treatment, as a common method was applied (*P* = 0.93). (Table 3).

**Figure 2:**
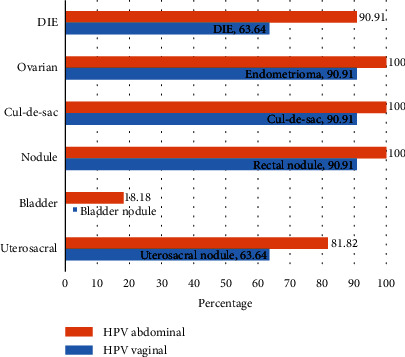
Comparison of endometriosis involvement between pelvic HPV- and vaginal HPV-infected cases.

In Figure 2, deep endometriosis and organ involvement were compared between pelvic HPV-infected women and vaginal HPV-infected women. Endometrioma, cul-de-sac involvement, and uterosacral nodules were more prevalent in pelvic HPV-positive patients. Unlike women with a vaginal HPV infection who had no bladder involvement, two patients with pelvic HPV showed bladder involvement. Based on the findings, endometrioma and deep endometriosis, including cul-de-sac involvement and uterosacral, rectal, and bladder nodules, were more common in pelvic HPV-positive cases.

In Table 4, the right and left sides of endometrioma and uterosacral involvement are compared between HPV-positive and HPV-negative patients. As shown in Table 4, there was no significant difference between the two groups, and the majority of patients showed both left- and right-sided involvement (*P* > 0.05).

HPV 6 and HPV 11, reported in seven and six patients, respectively, were the most common types of HPV in endometriosis patients. In Table 5, the HPV type is presented according to deep endometriosis status and organ involvement. HPV types 3, 6, 11, 16, 18, 35, 40, 51, 52, 53, and 68 were detected in 15%, 35%, 30%, 15%, 10%, 10%, 10%, 10%, 5%, 5%, and 10% of the patients, respectively.

## 4. Discussion

In the current study, the presence and genotypes of HPV were first investigated in confirmed cases of endometriosis, using HPV Direct Flow CHIP and PCR assay. Secondly, HPV-positive and HPV-negative groups were compared in terms of the baseline and clinical variables. The findings indicated that low-risk HPV was more prevalent in the study population. However, HPV was not associated with the severity of endometriosis, age, BMI, parity, disease duration since diagnosis, uterus size, history of surgery, urinary signs, menstrual status, or treatment method. Rectal nodule, uterosacral nodule, bladder nodule, and dysmenorrhea and dyspareunia indicate severity of endometriosis. Also, dysmenorrhea and dyspareunia were not significantly different between HPV-positive and HPV-negative groups, suggesting that HPV does not affect the severity of symptoms in endometriosis. According to studies conducted in Japan, China, and Turkey, there is a significant difference regarding the microbiota between women with and without endometriosis (18).

The prevalence of HPV infection in patients with endometriosis has been evaluated in the literature; nevertheless, the results vary between studies (19–21). In this study, the prevalence of HPV was estimated at 24.69% (20/81 patients), which is considerably lower than the prevalence rate reported in Brazil (82.8%) (21), but higher than statistics from Denmark (3%) (22) and Germany (11.3%) (20). Nevertheless, it is not yet clear whether the impact of ethnicity on the microbiota is related to genetic variability or culture. In studies by Jamdar et al. and Khodakarami et al., the prevalence of HPV in the Iranian population was estimated at 7.8-10.3% (23, 24). These results suggest that HPV is more common among patients with concluded endometriosis compared to the general population.

Additionally, in a study in Iran, Heidarpour et al. (25) investigated high-risk HPV infection in patients with endometriosis, using High Pure PCR to detect high-risk HPV. HPV was detected in 13 out of 40 endometriosis patients and five out of 49 cases in the control group. They concluded that high-risk HPV DNA is more likely to be found in endometriosis patients compared to the controls. Consistently, they found that age and parity were not associated with HPV infection in their study population. Besides, Heidarpour et al. (25) reported that HPV infection and endometriosis were inversely related to parity, and HPV infection was more common among infertile women.

The most common types of HPV in women with endometriosis were type 6 and type 11, which are both categorized as low risk. However, findings from other countries suggest different distributions for virus genotypes in endometriosis patients. In a study by Rocha et al. (21), HPV 16 (47.2%) was the most common type of HPV among endometriosis patients, followed by HPV 82 (13.9%) and HPV 6 (8.3%); in other words, the prevalence of high-risk HPV was higher in these patients. The observed differences might be related to the environment of the studied populations, genetics, or ethnic factors, which can be investigated in future studies.

Considering the pathophysiology of endometriosis, the possible role of infectious agents, such as HPV, in the development of endometriosis needs to be further examined. Genetic and epigenetic alterations, as well as the effects of infection on immunity and angiogenesis, can be caused by bacteria, viruses, and other microorganisms and lead to endometriosis lesions (26, 27). Currently, the association between HPV and endometriosis is subject to debate. In a study by Vestergaard et al. in Denmark, endometriosis lesions were examined regarding different types of HPV, herpes simplex virus 1 and 2 (HSV-1 and HSV-2), Epstein-Barr virus (EBV), cytomegalovirus (CMV), and polyomaviruses (SV40, JCV, BKV, KIV, WUV, and MCV). They found that DNA viruses in the endometrium and endometriosis lesions were not typical and rejected a virological cause for endometriosis. Moreover, they revealed that the endometrium may not be a proper site for HPV due to the low prevalence of this virus in deep tissues versus its high prevalence in more superficial parts of the reproductive tract (22).

In another study, Oppelt et al. reported that there is no definite information on whether HPV infection is associated with an increased incidence or risk of endometriosis. They showed that HPV might not be involved in the etiology of endometriosis, although it could promote transformation into a carcinoma (20). Overall, Heidarpour et al., Oppelt et al., and Rocha et al. reported higher rates of HPV detection for endometriosis lesions (20, 21, 25), whereas Vestergaard et al. found a low prevalence of HPV in endometriosis lesions; therefore, no significant association was suggested in their study (22).

The main limitation of this study was its cross-sectional design. Data on specific changes due to HPV would be more reliable if there was a control group for comparison. Also, considering the sexually transmitted nature of HPV, data on the sexual habits of the participants and the number of their partners were required, which can be challenging to collect. On the other hand, the strength of this study was the homogeneity of the participants' genetic and ethnic characteristics.

## 5. Conclusion

The prevalence of HPV was estimated at 24.69% in this study, and low-risk genotypes were dominant. The baseline characteristics of women did not differ significantly between infected and noninfected groups. Also, dysmenorrhea and dyspareunia were not significantly different between the groups, suggesting that HPV does not affect the severity of pain or disease in endometriosis patients. The association between HPV and endometriosis was not directly calculated, and it is recommended to conduct further cohort studies to conclusively confirm if endometriosis lesions are related to HPV infection.

## Figures and Tables

**Table 1:** Comparison of demographic, anthropometric, and clinical variables between HPV-infected and HPV-noninfected patients.

Variable	HPV infected	HPV noninfected	*P* value^∗^
Continuous variables (mean ± SD)			
Age (year)	35.65 ± 6.91	37.16 ± 4.95	0.29
BMI (kg/m^2^)	25.2 ± 3.59	25.95 ± 3.71	0.43
Gravidity (number)	1.2 ± 0.83	1.16 ± 1.08	0.89
Parity (number)	1.05 ± 0.69	1.02 ± 1.01	0.89
Endometrioma size (mm)	59.56 ± 23.51	55.86 ± 29.51	0.62
Uterus size (mm)	87.3 ± 12.48	91.75 ± 47.17	0.68
CA125 (U/ml)	67.73 ± 5.87	104.04 ± 169.53	0.71

Categorical variables (*n* (%))			
Irregular menses	9 (45)	25 (40.98)	0.75
Rectal nodule	19 (95)	53 (86.89)	0.32
Uterosacral nodule	14 (70)	52 (82.25)	0.13
Ovarian endometrioma	19 (95)	58 (95.08)	0.69
Bladder endometriosis	2 (10)	6 (9.84)	0.98
Infertility	3 (15)	28 (45.90)	0.014

^∗^Student's *t*-test for continuous variables and chi-square or Fisher's exact test for categorical variables.

**Table 2:** Comparison between HPV-infected and HPV-noninfected patients with regard to dysmenorrhea and dyspareunia status.

Variable		HPV infected *n* (%)	HPV noninfected *n* (%)	*P* value
Dysmenorrhea (*n* = 81)	Primary	5 (25)	2 (3.28)	0.003^∗^
	Secondary	15 (75)	59 (96.72)	
	VAS score	8.1 ± 1.25	7.9 ± 1.8	0.65^∗∗^
Dyspareunia (*n* = 68)		18 (90.0)	50 (81.96)	0.92
VAS score for dyspareunia		6.11 ± 2.59	5.84 ± 2.3	0.69^∗∗^

^∗^Fisher's exact test; ^∗∗^Student *t*-test.

**Table 3:** Comparison between HPV-infected and HPV-noninfected patients according to treatment type.

Variable		HPV infected *n* (%)	HPV noninfected *n* (%)	*P* value^∗^
Treatment type	No treatment	5 (25)	18 (29.51)	0.93
	Drug^∗∗^	11 (55)	28 (45.90)	
	Surgery	2 (10)	8 (13.11)	
	Both	2 (10)	7 (11.48)	

^∗^Fisher's exact test. ^∗∗^Hormonal treatment.

**Table 4:** Right or left side of endometrioma and uterosacral involvement in HPV-positive and HPV-negative patients.

Organ	HPV status		Right	Left	Bilateral	*P* value
Endometrium	Pelvic HPV	Yes	1 (9.09)	3 (27.27)	7 (63.64)	0.53
		No	15 (22.73)	12 (18.18)	39 (59.09)	
	Vaginal HPV	Yes	1 (10)	2 (20)	7 (70)	0.66
		No	15 (22.39)	13 (19.40)	39 (58.21)	
	Total HPV	Yes	2 (10.53)	4 (21.05)	13 (68.42)	0.44
		No	14 (24.14)	11 (18.97)	33 (56.90)	

Uterosacral	Pelvic HPV	Yes	3 (33.33)	1 (11.11)	5 (55.56)	0.54
		No	11 (19.30)	4 (7.02)	42 (73.68)	
	Vaginal HPV	Yes	2 (28.57)	1 (14.29)	4 (57.14)	0.64
		No	12 (20.34)	4 (6.78)	43 (72.88)	
	Total HPV	Yes	4 (28.57)	2 (14.29)	8 (57.14)	0.37
		No	10 (19.23)	3 (5.77)	39 (75.00)	

**Table 5:** HPV type according to DE status and organ involvement.

Variable			HPV type (number)
HPV vaginal	DE status	DE+	3, 6, 16, 18, 51, 53, 68
		DE-	6 (3), 11, 18, 40, 68
	Organ involvement	Rectal nodule	3, 6 (4), 11, 16, 18, 40, 51, 53, 68 (2)
		Uterosacral nodule	3, 6 (3), 11, 16, 18, 40, 51, 53, 68
		Cul-de-sac involvement	3, 6 (4), 11, 16, 18 (2), 40, 51, 53, 68 (2)
		Ovarian endometrioma	3, 6 (2), 11, 16, 18 (2), 40, 51, 53, 68
		Bladder endometriosis	

Pelvic HPV	DE status	DE+	6 (2), 11 (5), 16 (2), 35 (2), 40, 51, 52
		DE-	6
	Organ involvement	Rectal nodule	6 (3), 11 (5), 16 (2), 35 (2), 40, 51, 52
		Uterosacral nodule	6 (2), 11 (4), 16 (2), 35 (2), 40, 51
		Cul-de-sac involvement	6 (3), 11 (5), 16 (2), 35 (2), 40, 51, 52

## Data Availability

The data that support the findings of this study are available from the corresponding author upon reasonable request.
